# Behavior Change Techniques Incorporated in Fitness Trackers: Content Analysis

**DOI:** 10.2196/12768

**Published:** 2019-07-23

**Authors:** Gladys Lai Cheng Chia, Angelika Anderson, Louise Anne McLean

**Affiliations:** 1 Monash University Clayton Australia; 2 The University of Waikato Hamilton New Zealand

**Keywords:** behavioral medicine, self-management, fitness tracker, physical activity, sedentary behavior

## Abstract

**Background:**

The use of fitness trackers as tools of self-management to promote physical activity is increasing. However, the content of fitness trackers remains unexplored.

**Objective:**

The aim of this study was to use the Behavior Change Technique Taxonomy v1 (BCTTv1) to examine if swim-proof fitness trackers below Aus $150 (US$ 105) incorporate behavior change techniques (BCTs) that relate to self-management strategies to increase physical activity and reduce sedentary behavior and to determine if content of the fitness trackers correspond to physical activity guidelines.

**Methods:**

A total of two raters used the BCTTv1 to code 6 fitness trackers that met the inclusion criteria. The inclusion criteria were the ability to track activity, be swim proof, be compatible with Android and Apple operating systems, and cost below Aus $150.

**Results:**

All fitness trackers contained BCTs known to promote physical activity, with the most frequently used BCTs overlapping with self-management strategies, including goal setting, self-monitoring, and feedback on behavior. Fitbit Flex 2 (Fitbit Inc) contained the most BCTs at 20. Huawei Band 2 Pro (Huawei Technologies) and Misfit Shine 2 (Fossil Group) contained the least BCTs at 11.

**Conclusions:**

Fitness trackers contain evidence-based BCTs that overlap with self-management strategies, which have been shown to increase physical activity and reduce sedentary behavior. Fitness trackers offer the prospect for physical activity interventions that are cost-effective and easily accessed by a wide population.

## Introduction

Regular physical activity has many health benefits, but many individuals are still not meeting the minimum guidelines for physical activity recommended by the World Health Organization and various public health organizations [1]. The inadequate level of physical activity to meet present physical activity guidelines has been referred to as physical inactivity [2-4], and it is increasingly being recognized as a major problem in global health [5,6]. Physical inactivity is considered to be the fourth leading risk factor for mortality, with an estimated contribution of 3.2 million deaths worldwide [4]. Sedentary behavior is also recognized as a contributing factor to global health issues [7], and it is defined as “any waking behavior characterized by an energy expenditure ≤1.5 metabolic equivalent tasks (METs) while in a sitting or reclining posture” [8]. Research indicates that prolonged sedentary behavior is linked to endothelial dysfunction [9], associated with increased risk of type 2 diabetes [10,11] and mortality because of cardiovascular disease [12,13]. Although being sedentary can contribute to higher levels of inactivity, it is not synonymous with physical inactivity. It is also not the inverse of physical activity. For example, individuals who run for 40 min in the morning and then spend the rest of their day sitting are considered to be physically active (having met the minimum guidelines for physical activity) and sedentary, whereas those who spend their day standing and do not engage in other physical activity are considered to be physically inactive but not sedentary. Health organizations have recognized the need to address physical activity and sedentary behavior as separate constructs, and they have included sedentary behavior recommendations along with physical activity guidelines [14-16].

Interventions to decrease both physical inactivity and sedentary behavior are much needed. They should be affordable and accessible across population groups, which is particularly pertinent, given the higher prevalence of physical inactivity and sedentary behavior documented in those with less education and lower socioeconomic status [17,18]. Behavioral interventions that include self-management strategies to promote physical activity are emerging. Systematic reviews and meta-analyses have found that behavioral change techniques related to self-management strategies were effective in increasing physical activity in young and middle-age adults [19-22], older adults [23], and overweight and obese adults [24,25]. Furthermore, techniques related to self-management were found to be linked to maintenance of physical activity behavior [19], and these were also effective in reducing sedentary behavior [26]. The use of wearable fitness trackers as tools for self-management in physical activity interventions [27-30] is growing. These fitness trackers are typically worn on the body and are able to monitor and track statistics, such as distance walked or ran, number of steps taken, and calorie expenditure. Some fitness trackers are able to offer coaching and feedback during activities and provide prompts to engage in activity. Studies that examined the use of fitness trackers have shown them to be effective in increasing physical activity with adults who are inactive [31], sedentary [32], overweight and obese [33,34], patients with chronic illness, such as cancer [35-38], and older adults [39-42]. Behavior change techniques (BCTs) are observable, replicable, and irreducible components of interventions that can bring about behavioral change [43,44], including increased physical activity [19,45,46]. Taxonomies to identify BCTs have been developed and refined over the years, with the latest being the Behavior Change Technique Taxonomy v1 (BCTTv1). The BCTTv1 contains 93 distinct BCTs, and it can be used across behaviors and disciplines [47]. As individual BCTs are seldom applied in isolation, the combination of BCTs has been a topic of focus in recent studies. In terms of interventions to promote physical activity, it was found that the effectiveness of such interventions was enhanced when a self-monitoring BCT was combined with other self-management–related BCTs, such as goal setting, feedback on performance, and review of behavioral goals [20,48]. This combination of self-management–related BCTs has also been posited to contribute to maintenance of behavior change [49,50]. Another combination of BCTs that supports maintenance of change in physical activity includes BCTs that address capability, such as instruction or demonstration of behavior, and provide information about the significance of behavior change [25]. Furthermore, a meta-analysis that examined the effects of physical activity interventions found that a combination of at least 3 BCT clusters out of the 16 specific BCT clusters from the BCTTv1 was required within physical activity interventions to yield significant effects [51]. With the increasing use of fitness trackers as self-management tools to promote physical activity and reduce sedentary behavior, the lack of studies investigating their use of evidence-based techniques for behavioral change is surprising. Findings from the few studies that have investigated the use of BCTs in fitness trackers have shown that fitness trackers contained evidenced-based BCTs that supported users to increase physical activity [52-55]. Most of these BCTs were based around self-management strategies, mostly targeting physical activity rather than sedentary behavior [54]. Some aspects not addressed by these previous studies were the affordability of activity trackers and whether they corresponded to physical activity guidelines. Affordable and accessible interventions are much needed, particularly for individuals from low socioeconomic backgrounds who have been reported to be more inactive and sedentary [17,18]. This study assessed fitness trackers below Aus $150 (US$ 105) that are suitable for both land and water activities (henceforth swim proof), as identified on their official product websites, to address affordability and accessibility in terms of a wider coverage of activity types. The objective of this study was to examine if swim-proof fitness trackers below Aus $150 (1) incorporate BCTs that relate to self-management strategies, such as stimulus control, self-monitoring, and self-delivery of consequences, which have been linked to increased physical activity and reduced sedentary behavior, and the objective of this study was to (2) determine if they correspond to physical activity guidelines, particularly the accumulation of at least 150 min of moderate-to-vigorous physical activity per week and the reduction of sedentary behavior by minimizing the amount of prolonged sitting.

## Methods

### Search Strategy

Various brands of fitness trackers were identified on the basis of listings from 4 websites (CNET, TechRadar, Wareable, and AllThingsWaterproof). A list of their corresponding webpages about fitness trackers was created on January 9, 2018. The official product websites for each brand of fitness trackers were reviewed to identify the various models of fitness trackers pertaining to each brand. Each identified fitness tracker was assessed for the following inclusion and exclusion criteria through reviewing the specifications of each fitness tracker on its official product website.

### Inclusion Criteria

Inclusion criteria for the fitness trackers were the following: (1) be able to track or monitor activity (eg, number of steps taken, minutes spent in swimming), (2) be swim proof, (3) be compatible with both Android and Apple operating systems, and (4) cost below Aus $150.

### Coding Procedure

A total of two raters, the first author (GC) and an independent researcher (PP), wore each of the fitness trackers for a week and downloaded and used companion apps of each fitness tracker on a smartphone. The fitness trackers and their companion apps were coded using the BCTTv1 coding manual, which contains labels, descriptions, and examples of each BCT. The raters also read the manual of each fitness tracker to ensure that no functions of the fitness tracker were left unnoticed and unassessed. Each BCT was coded using a dichotomous score of either 0 (not present) or 1 (present). Any coding disagreements were discussed between the two raters until an agreement was reached. To support study objectives, BCTs were only coded when they were applied to target behaviors of increasing physical activity or reducing sedentary behavior. BCTs that targeted other behaviors (eg, diet or sleep) were not coded. Before assessing the fitness trackers and their companion apps, the main author completed the Web-based BCTTv1 training through the official website and trained the other rater to use the BCTTv1 taxonomy. After this, a test for calibration was conducted, where the mobile app Runkeeper was coded using the BCTTv1 taxonomy. This app was chosen as it was found to contain the highest number of BCTs in a review of apps to promote physical activity in adults [56]. Furthermore, by using an app, the two raters were able to access the app and conduct the calibration process concurrently. Any ambiguous descriptors or definitions from the BCTTv1 taxonomy were discussed between the two raters until an agreement was reached during the calibration process.

### Materials

The 6 fitness trackers that were included in this study were the Fitbit Flex 2 (Fitbit Inc), Huawei Band 2 Pro (Huawei Technologies), Misfit Shine 2 (Fossil Group), Moov Now (Moov Inc), Nokia Go (Withings), and Polar A300 (Polar Electro). The companion app of each fitness tracker was downloaded to an Apple iPhone 8 and a Samsung J5 Pro smartphone. Record forms were used to document the results from the data analysis of each fitness tracker. Each form included a table containing labels, definitions, and examples of each BCT from the BCTTv1 coding manual, as well as a 0 and 1 against each BCT for the raters to mark the absence or presence of the BCT.

### Data Analysis

Descriptive statistics were used to summarize the BCTTv1 ratings of the fitness trackers. Interrater reliability was calculated by dividing the number of agreements plus disagreements and multiplying by a hundred. To assess the relationship between the cost of fitness trackers and the number of BCTs incorporated, a Pearson correlation coefficient was computed via SPSS Version 26 (IBM), using an alpha of .05 to determine statistical significance.

## Results

### Fitness Tracker Selection

**Figure 1:**
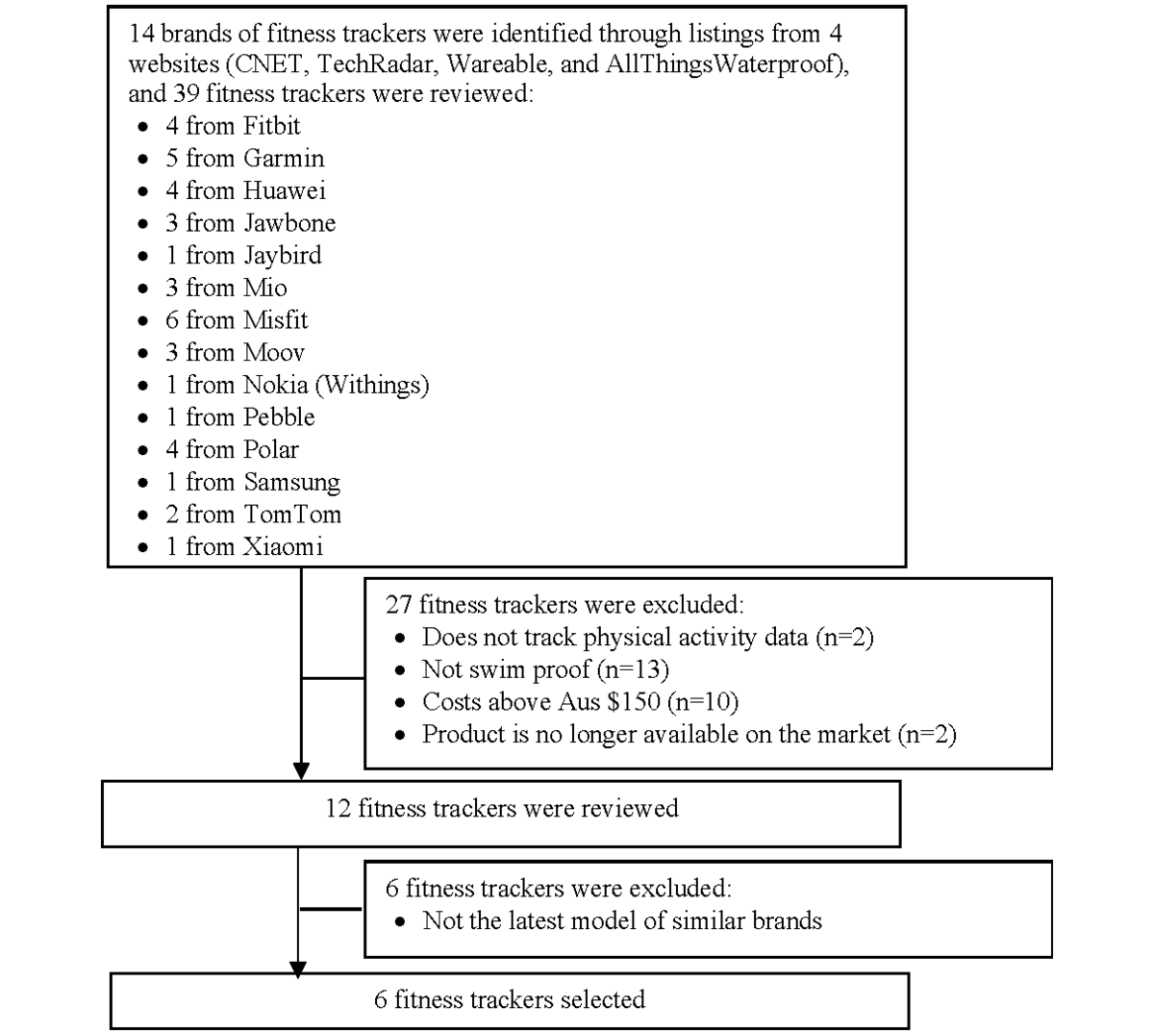
Flow diagram describing the fitness tracker search and selection process.

A total of 39 fitness trackers were identified across 14 brands as shown in Figure 1. These 39 fitness trackers were assessed by reviewing their specifications on their official product website, and 12 of them were considered eligible for inclusion. Of the eligible 12 fitness trackers, 6 fitness trackers were variants of others from similar brands. As such, only the latest model of each brand of fitness tracker was retained, leaving a total of 6 fitness trackers for BCT analysis.

### Presence of Behavior Change Techniques

The definition of each BCT is provided in Multimedia Appendix 1. The number and type of BCTs included in each fitness tracker were evaluated, and these are summarized in Tables 1 and 2. The interrater reliability for evaluating the presence of BCTs measured by percent of agreement was 100%. Disagreements were resolved through discussion. The cost, number of BCT clusters as identified by the BCTTv1 classification, and the number of self-management and nonself-management BCTs incorporated in each fitness tracker are outlined in Table 1.

**Figure 2:**
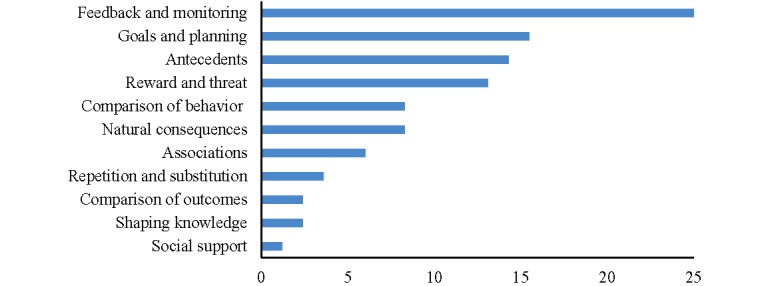
Mean percentage of behavior change technique clusters incorporated across all fitness trackers.

The average number of BCT clusters included in each fitness tracker was 12, ranging from 6 to 9. The Fitbit Flex 2 and Nokia Go had the highest number of BCT clusters (n=9), followed by Huawei Band 2 Pro (n=8). Misfit Shine 2 had the lowest number of BCT clusters (n=6). The most common BCT cluster incorporated across all fitness trackers was *goals and planning* (mean BCTs incorporated=25%), followed by *feedback and monitoring* (mean BCTs incorporated=15.5%), *antecedents* (mean BCTs incorporated=14.3%), and *reward and threat* (mean BCTs incorporated=13.1%), as shown in Figure 2. The average number of BCTs per fitness tracker was 14, ranging from 11 to 20. The Fitbit Flex 2 had the highest number of BCTs (n=20), followed closely by Nokia Go (n=19). The Huawei Band 2 Pro and Misfit Shine 2 had the lowest number of BCTs (n=11). The Fitbit Flex 2 incorporated the most number of BCTs related to self-management strategies (n=14), followed by Nokia Go (n=12). Huawei Band 2 Pro and Polar A300 contained the least number of BCTs related to self-management strategies (n=9).

The names and types of BCTs incorporated in each fitness tracker are outlined in Table 2.

A total of 6 BCTs out of the total 93 were present in every fitness tracker. These 6 BCTs were *discrepancies between current behavior and goal, feedback on behavior, self-monitoring of behavior, feedback on outcomes of behavior*,*adding objects to the environment*, and *body changes*. A total of 8 BCTs were present in 50% or more of the fitness trackers. These were *goal setting of behavior* (n=5), *goal setting of outcomes* (n=3), *self-monitoring of outcomes of behavior* (n=3), *information about health consequences* (n=4), *social comparison* (n=5), *prompts and cues* (n=5), *social reward* (n=4), and *social incentive* (n=3). The BCTs that were present in fewer than 50% of the fitness trackers were *action planning* (n=1), *social support unspecified* (n=1), *instruction on how to perform behavior* (n=2), *monitoring of emotional consequences* (n=1), *information about emotional consequences* (n=2), *demonstration of behavior* (n=2), *behavioral practice and rehearsal* (n=1), *habit formation* (n=1), *graded tasks* (n=1), *credible source* (n=2), *incentive outcome* (n=1), and *reward outcome* (n=2). A Pearson correlation coefficient was computed to assess the relationship between the cost of fitness trackers and the number of BCTs incorporated. There was a nonsignificant correlation (r=0.21, n=6, *P*=.69) between cost of fitness trackers and the number of BCTs incorporated.

**Table 1:** Cost and the number of behavior change techniques incorporated in each fitness tracker.

Fitness tracker	Recommended retail price (Aus $)	BCT^a^ clusters included (% of total 16 clusters), n (%)	BCTs included (% of total 93 BCTs), n (%)	BCTs related to self-management strategies, n	BCTs not related to self-management strategies, n
Fibit Flex 2	149.95	9 (56)	20 (21)	14	6
Polar A300	149	7 (44)	12 (13)	9	3
Misfit Sine 2	107.77	6 (38)	11 (12.8)	10	1
Huawei Band 2 Pro	98	8 (50)	11 (12.8)	9	2
Nokia Go	89.95	9 (56)	19 (20)	12	7
Moov Now	78.26	7 (44)	13 (14)	10	3

^a^BCT: behavior change technique.

**Table 2:** Behavior change techniques’ effect in increasing physical activity included in each fitness tracker.

Behavior change techniques			Fitbit Flex 2	Huawei Band 2 Pro	Misfit Shine 2	Moov Now	Nokia Go	Polar A300
**Behavior change techniques** **related to self-management strategies**								
	**Antecedents**							
		Adding objects to the environment	✓^a^	✓	✓	✓	✓	✓
		Body changes	✓	✓	✓	✓	✓	✓
		Prompts/ or cues	✓	✓	✓	—^b^	✓	✓
		Goal setting (behavior)	✓	✓	—	✓	✓	✓
		Goal setting (outcome)	✓	—	✓	—	✓	—
		Graded tasks	—	—	—	✓	—	—
	**Self-monitoring and self-evaluation**							
		Self-monitoring of behavior	✓	✓	✓	✓	✓	✓
		Self-monitoring of outcome(s) of behavior	✓	—	✓	—	✓	—
		Feedback on behavior	✓	✓	✓	✓	✓	✓
		Feedback on outcome(s) of behavior	✓	✓	✓	✓	✓	✓
		Discrepancy between current behavior and goal	✓	✓	✓	✓	✓	✓
	**Self-delivery of consequences**							
		Social reward	✓	✓	—	✓	✓	✓
		Social incentive	✓	—	—	✓	✓	—
		Reward (outcome)	✓	—	✓	—	—	—
		Incentive (outcome)	✓	—	—	—	—	—
**Behavior change techniques not related to self-management strategies**								
	Information about health consequences		✓	✓	—	—	✓	✓
	Information about emotional consequences		✓	—	—	—	✓	—
	Instruction on how to perform a behavior		✓	—	—	✓	—	—
	Demonstration of the behavior		✓	—	—	✓	—	—
	Behavior practice and rehearsal		—	—	—	—	✓	—
	Action Planning		—	—	—	—	✓	—
	Habit Formation		—	—	—	—	✓	—
	Credible Source		✓	✓	—	—	—	—
	Social Comparison		✓	—	✓	✓	✓	✓
	Social Support		—	—	—	—	✓	—
	Monitoring of emotional consequences		—	—	—	—	—	✓
Total			20	11	11	13	19	12

^a^Indicates behavior change technique found in the fitness tracker.

^b^Indicates behavior change technique not found in the fitness tracker.

### Presence of Behavior Change Techniques Related to Self-management Strategies

All fitness trackers incorporated components of self-management strategies to target participation in physical activity. These self-management components included goal setting, self-monitoring, and self-evaluation of activity in relation to goals, providing feedback of progress toward goals, and providing rewards on meeting goals.

#### Antecedents

Antecedent-based self-management strategies involve the manipulation stimuli, such as the environment or motivating operations, to increase the desired behavior [57]. All fitness trackers incorporated antecedent-based BCTs. Each fitness tracker device was a stimulus that was added to the environment, and 5 fitness trackers (Fitbit Flex 2, Huawei Band 2 Pro, Misfit Shine 2, Nokia Go and, Polar A300) provided prompts as reminders to be physically active. Prompts were inactivity alerts through vibration and flashing lights on the fitness tracker device, as well as push notifications on the app to go for planned activities or to hit target goals. All fitness trackers incorporated BCTs of goal setting to increase physical activity. Goal setting was achieved using the companion apps of the fitness trackers. A total of 5 fitness trackers (Fitbit Flex 2, Huawei Band 2 Pro, Moov Now, Nokia Go, and Polar A300) incorporated *goal setting of behavior*, such as setting the number of steps taken per day or distance covered. A total of 3 fitness trackers (Fitbit Flex 2, Misfit Shine 2, and Nokia Go) incorporated *goal setting of outcomes*, which allowed users to set a target weight. Only Moov Now incorporated *graded tasks*, in which a new goal of increased steps was suggested on the app when the previous goal had been achieved.

#### Self-Monitoring and Self-Evaluation

Self-monitoring related BCTs were incorporated in all fitness trackers. All fitness tracker apps automatically tracked the number of steps taken and allowed users to manually record other nonstep physical activities, such as swimming, cycling, and weight training. A total of 3 fitness trackers (Fitbit Flex 2, Misfit Shine 2, and Nokia Go) provided the functionality for users to record their weight using the companion apps.

Feedback on current behavior, such as duration of physical activity, number of steps taken, and distance covered, as well as number of calories burned, were presented on all fitness tracker apps. All fitness trackers also supported self-evaluation by presenting discrepancies between goals and results of current behavior through the use of visual indicators, such as bar charts, progress bars, and doughnut charts. These charts were often shaded or color coded to indicate proximity to goals, and these charts were presented on all fitness tracker apps, but they were presented only on 3 of the 6 fitness tracker devices (Huawei Band 2 Pro, Nokia Go, and Polar A300).

#### Self-Delivery of Consequences

All fitness trackers provided rewards to strengthen the target behavior of increasing physical activity. A total of 5 fitness trackers (Fitbit Flex 2, Huawei Band 2 Pro, Moov Now, Nokia Go, and Polar A300) incorporated *social reward*, which comprised congratulatory notifications and badges presented through the app when behavioral goals (eg, number of steps) were met. A total of 3 fitness trackers (Fitbit Flex 2, Moov Now, and Nokia Go) incorporated *social incentive*, which comprised presenting badges that users can unlock or earn on the apps. A total of 2 fitness trackers (Fitbit Flex 2 and Misfit Shine 2) incorporated *reward outcome*, which comprised congratulatory messages through the app, as well as vibration and animating lights on the device when outcomes of goals (eg, points earned through physical activity, calories burned) were met. Only the Fitbit Flex 2 incorporated *incentive outcome*, which comprised using text to inform users that they will receive celebratory messages when their goal outcomes (eg, calories burned) were met. Notably, self-management components found in Fitbit Flex 2, Huawei Band 2 Pro, Misfit Shine 2, and Polar A300 also targeted inactivity. This was achieved through setting a reminder to move after an hour of inactivity, which can also be considered as a form of goal setting and prompting. The devices vibrated and provided visual prompts as indicators of inactivity. The Fitbit Flex 2 and Polar A300 also provided feedback through visual charts of inactivity on the app, identifying the specific hour in the day where inactivity occurred.

### Presence of Other Behavior Change Technique to Increase Physical Activity

A total of 11 BCTs that were not related to self-management strategies were found to be incorporated across the fitness trackers. A total of 4 fitness trackers (Fibit Flex 2, Huawei Band 2 Pro, Nokia Go, and Polar A300) provided *information about health consequences* through textual information on the app (eg, “put on your running shoes…work up a sweat…it can improve cardiovascular and respiratory health”). A total of 2 fitness trackers (Fitbit Flex 2 and Nokia Go) incorporated *information about emotional consequences*, presented as textual information on the app (eg, “…boost self-esteem and emotional state”). A total of 2 fitness trackers (Fitbit Flex 2 and Moov Now) incorporated the BCT *instruction on how to perform behavior* and *demonstration of behavior*, which were presented through videos on the app showing movements and describing the steps to undertake the physical activity. A total of 2 fitness trackers (Fitbit Flex 2 and Huawei Band 2 Pro) incorporated *credible source*, presented as textual information on the app (eg, “the American heart association recommends…”). All fitness trackers, except the Huawei Band 2 Pro, incorporated social comparison, using leaderboards of steps accumulated. Only the Nokia Go incorporated the BCTs *behavioral practice and rehearsal*, *action planning*, *habit formation*, and *social support* by setting alerts as prompts for action. Only the Polar A300 incorporated *monitoring of emotional consequences* through rating of emotions using emoticons on the app.

### Correspondence to Physical Activity Guidelines

All 6 fitness trackers incorporated BCTs to increase physical activity, but only 2 fitness trackers (Fitbit Flex 2 and Moov Now) aligned with public health physical activity guidelines by including active minutes as a physical activity goal. The Huawei Band 2 Pro, Misfit shine 2, Nokia Go, and Polar A300 centered on the number of steps achieved daily as physical activity goals. A total of 2 fitness trackers (Fitbit Flex 2 and Polar A300) incorporated BCTs to reduce sedentary behavior, which was implemented through the BCT *information about health consequences* (eg, “sitting for long periods is bad for your blood circulation, especially in your legs” and “moving regularly breaks up sedentary time and can improve your well-being”). The Huawei Band 2 Pro, Misfit shine 2, Moov Now, and Nokia Go did not specifically target sedentary behavior.

## Discussion

### Principal Findings

This study aimed to evaluate the use of BCTs in fitness trackers that are swim proof and cost less than Aus $150 (US$ 105). Overall, all fitness trackers incorporated more than 3 BCT clusters, which has been shown to produce significant effects in physical activity interventions [51]. The Fitbit Flex 2 and Nokia Go incorporated the most BCT clusters. There was a nonsignificant correlation between the cost of fitness trackers and the number of BCTs incorporated. The Fitbit Flex 2, which costs Aus $149.95 (US$ 105, had the most BCTs (coded at 20), followed by the Nokia Go, at a cost of Aus $89.95 (US$ 63) with 19 BCTs. In comparison, the Misfit Shine 2 Pro, which costs Aus $107.77 (US$ 75), incorporated the least number of BCT clusters and BCTs, at 6 and 11, respectively. The findings indicated that the cost of fitness trackers does not necessarily reflect or is associated with the number of BCTs incorporated in them. The BCTs that were frequently incorporated across the 6 fitness trackers were mostly related to self-management strategies, such as goal setting, self-monitoring, self-evaluation (eg, feedback, provision of discrepancies between current behavior and goal), prompts and cues, and rewards (eg, social reward) [57]. This finding is similar to those from previous studies of fitness trackers [52-54] and apps [56,58] targeting physical activity, and they also overlap with reviews of BCTs that were rated as important by users of fitness trackers [55]. Notably, all 6 fitness trackers in this study incorporated a combination of 9 or more BCTs that were related to self-management. However, similar to the study by Lyons et al [52], this study also found that other effective BCTs were rarely incorporated in fitness trackers. Only Nokia Go contained the BCTs *behavior practice and rehearsal* and *social support*, through the use of prompts, that have been found to be effective in increasing physical activity for adults with obesity [24] and cardiovascular disease [45]. This indicates that certain fitness trackers may be more effective for particular population groups because of the types of BCTs incorporated in them. The combinations of informational and instructional BCTs that have been found to support maintenance of change in physical activity were only observed in the Fitbit Flex 2 fitness tracker. The Fitbit Flex 2 companion app provided links to a separate app called Fitbit Coach that contained a library of videos to instruct and model the way to perform various workouts. The Fitbit Flex 2 companion app also provided textual information about health and emotional benefits of physical activity and sedentary behavior. It seems that manufacturers of fitness trackers are more focused on functional features (eg, recording data, providing prompts) compared with informational and instructional features. It could be that certain BCTs, such as features that support self-management through recording behavioral data, evaluating and providing feedback about behavior and providing prompts, are better suited to delivery via wearable technology. There is some support for this contention in this study in the finding that informational and instructional BCTs were often presented on the companion apps instead of the device. A recent study also found that providing instructions was one of the most frequently implemented BCTs (17 of 25 apps) in mobile apps that aimed to improve physical activity and reduce sedentary behavior [59]. These findings are not surprising, as longer information and instructions might be more suited to technologies with larger reading screens or audio outputs, such as computers, tablets, and smartphones. Taken together, these findings highlight the importance of delivering BCTs through appropriate technological channels and leveraging and integrating different technologies to create a more holistic intervention for promoting physical activity.

Given that the purpose of fitness trackers is to promote physical activity, it is remarkable that not all fitness trackers assessed in this study aligned with public health physical activity guidelines. Physical activity guidelines for adults include engagement of at least 150 min of moderate-intensity physical activity per week, performed in bouts of at least 10 min duration [4,14,60]. Only the Fitbit Flex 2 and Moov Now aligned with public health physical activity guidelines by including active minutes as a physical activity goal. Active minutes were calculated only if activities above 3 METs were detected continuously for 10 min at a time. Other fitness trackers focused on the number of steps achieved daily. The tracking of steps is a prominent part of data collection for most of the fitness trackers in this study. This was expected, as steps are a fundamental part of daily living, which might be more easily measured. However, the sole use of steps to measure physical activity may be insufficient. The tracking of step counts does not provide information about the intensity of the activity, which has been indicated to be more beneficial to health than number of steps taken [61]. Moreover, there has been a change in the fitness culture over the past decade, in which the trend for physical activity has been moving away from step-based activities to spinning (indoor cycling), dance workouts (eg, Zumba), and body weight training [62-64], as well as water sports, such as stand-up paddle boarding and kitesurfing. Although most fitness trackers allow for self-monitoring of physical activity that is not based on steps, these data have not been directly incorporated into physical activity goals. Given the findings from previous studies that goal setting is an effective BCT to increase physical activity [25,48] and reduce sedentary behavior [65], it would be optimal to integrate non-step-based activities into physical activity goals, as has been achieved by Fitbit Flex 2 and Moov Now, through the inclusion of active minutes as a goal. Regarding guidelines to minimize sedentary behavior by minimizing the amount of prolonged sitting [14-16], only the Fitbit Flex 2 and Polar A300 provided BCTs that directly targeted sedentary behavior in the form of providing *information about health consequences*. The Fitbit Flex 2, Huawei Band 2 Pro, Misfit Shine 2, and Polar A300 provided prompts to move after detecting inactivity over 1- or 2-hour periods. However, these prompts were based on a lack of steps detected within that period, instead of detecting sedentary behavior that has been defined as engaging in a sitting or reclining posture, with an energy expenditure less than or equal to 1.5 METs [8]. The authors of a previous study have coded the prompts of inactivity as targeting a reduction of sedentary behavior [54]. However, this study adhered to the definition of sedentary behavior by the Sedentary Behaviour Research Network [8] and took into account the distinction between physical inactivity and sedentary behavior in the coding of BCTs. Furthermore, studies have noted that interventions, which promote physical activity, do not necessarily have an effect on reducing sedentary behavior, and techniques that primarily aimed to change sedentary behavior were more effective in reducing sedentary behavior [65]. Despite literature showing that both physical activity and sedentary behavior are important to good health, it seemed that most developers of fitness trackers assessed in this study have focused on achieving sufficient amount of physical activity, but they have placed less emphasis on directly reducing sedentary behavior. It could be that the technology required to detect sitting and reclining postures is too costly to be incorporated into fitness trackers within the price range of Aus $150 and below and that a more cost-effective workaround to target sedentary behavior is to frequently prompt users to take more steps. Overall, the findings from this study show that fitness trackers below Aus $150 contain evidence-based BCTs known to promote physical activity and reduce sedentary behavior. The majority of BCTs implemented targeted physical activity, and the most frequently used BCTs overlapped with self-management strategies. This suggests that fitness trackers offer the prospect for physical activity interventions that are cost effective and easily accessed by a wide population. Opportunities to improve the effectiveness of fitness trackers include incorporating BCTs that facilitate maintenance of behavior change in physical activity, such as instruction or demonstration of behavior, and providing information on the consequences of behavior [24], as well as incorporating BCTs to target sedentary behavior. Furthermore, public health physical activity guidelines ought to be used to inform the type of goals that are incorporated into fitness trackers.

Although this study highlights that low cost fitness trackers contain evidence-based BCTs known to promote physical activity, it is worth noting that only 1 fitness tracker contained BCTs effective for special population groups, such as adults with obesity and chronic illnesses. Further work is needed to determine if these low cost fitness trackers are sufficient as standalone interventions, especially for these special population groups.

### Strengths and Limitations

The most recent taxonomy of behavioral change techniques was used to assess the content of the fitness trackers and their companion apps, and it compared the BCTs to those that have been found to be successful at improving physical activity, thus adding to the evidence base for the use of fitness trackers to support interventions to promote physical activity. This study focused on fitness trackers that are swim proof and cost less than Aus $150 to broaden the reach of fitness trackers to the community in terms of preferred and accessible physical activity, as well as affordability. A limitation is that the ratings may not be representative of the most current version of the fitness trackers assessed in this study because of frequent updates of fitness trackers and their associated apps. A further limitation is that this study only included fitness trackers under Aus $150 and did not cover the entire range of fitness trackers available. The small number of fitness trackers places a limitation on the calculation of Pearson correlation coefficient to assess the relationship between the cost of fitness trackers and the number of BCTs incorporated.

## Appendix

Multimedia Appendix 1 Description of behavior change techniques included in each fitness tracker.

## Abbreviations

BCT: Behavior Change Technique
BCTTv1: Behavior Change Technique Taxonomy v1
MET: Metabolic Equivalent Tasks
