# Supplementary material for: Behavior Change Techniques Incorporated in Fitness Trackers: Content Analysis
Source: JMIR Mhealth Uhealth. 2019 Jul 23;7(7):e12768. doi: 10.2196/12768 (PMC6683653; doi:10.2196/12768)
Supplement: Multimedia Appendix 1 [file mhealth_v7i7e12768_app1.docx]

Appendix

Table 1. Description of BCTs included in each fitness tracker

| *No.* | *Label* | *Definition* | *Fitbit Flex 2* | *Huawei Band 2 Pro* | *Misfit Shine 2* | *Moov Now* | *Nokia Go* | *Polar A300* |
| --- | --- | --- | --- | --- | --- | --- | --- | --- |
|  | *Goals and planning* | | | | | | | |
| 1.1 | Goal setting (behaviour) | Set or agree on a goal defined in terms of the behaviour to be achieved. | 1 | 1 |  | 1 | 1 | 1 |
| 1.2 | Problem solving | Analyse, or prompt the person to analyse, factors  influencing the behaviour and generate or select strategies that include overcoming barriers and/or increasing facilitators. |  |  |  |  |  |  |
| 1.3 | Goal setting (outcome) | Set or agree on a goal defined in terms of a positive outcome of wanted behaviour. | 1 |  | 1 |  | 1 |  |
| 1.4 | Action planning | Prompt detailed planning of performance of the behaviour (must include at least one of context, frequency, duration and intensity). Context may be environmental (physical or social) or internal (physical, emotional or cognitive). |  |  |  |  | 1 |  |
| 1.5 | Review behaviour goal(s) | Review behaviour goal(s) jointly with the person and consider modifying goal(s) or behaviour change strategy in light of achievement. |  |  |  |  |  |  |
| 1.6 | Discrepancy between current behaviour and goal | Draw attention to discrepancies between a person’s current behaviour (in terms of the form, frequency, duration, or intensity of that behaviour) and the person’s previously set outcome goals, behavioural goals or action plans (goes beyond self-monitoring of behaviour). | 1 | 1 | 1 | 1 | 1 | 1 |
| 1.7 | Review outcome goal(s) | Review outcome goal(s) jointly with the person and consider modifying goal(s) in light of achievement. |  |  |  |  |  |  |
| 1.8 | Behavioural contract | Create a written specification of the behaviour to be performed, agreed on by the person, and witnessed by another. |  |  |  |  |  |  |
| 1.9 | Commitment | Ask the person to affirm or reaffirm statements indicating commitment to change the behaviour. |  |  |  |  |  |  |
|  | *Feedback and monitoring* | | | | | | | |
| 2.1 | Monitoring of behaviour by others without feedback | Observe or record behaviour with the person’s knowledge as part of a behaviour change strategy. |  |  |  |  |  |  |
| 2.2 | Feedback on behaviour | Monitor and provide informative or evaluative feedback on performance of the behaviour (e.g. form, frequency, duration, intensity). | 1 | 1 | 1 | 1 | 1 | 1 |
| 2.3 | Self-monitoring of behaviour | Establish a method for the person to monitor and record their behaviour(s) as part of a behaviour change   strategy. | 1 | 1 | 1 | 1 | 1 | 1 |
| 2.4 | Self-monitoring of outcome(s) of behaviour | Establish a method for the person to monitor and record the outcome(s) of their behaviour as part of a behaviour change strategy. | 1 |  | 1 |  | 1 |  |
| 2.5 | Monitoring outcome(s) of behaviour by others without feedback | Observe or record outcomes of behaviour with the person’s knowledge as part of a behaviour change strategy. |  |  |  |  |  |  |
| 2.6 | Biofeedback | Provide feedback about the body (e.g. physiological or biochemical state) using an external monitoring device as part of a behaviour change strategy |  |  |  |  |  |  |
| 2.7 | Feedback on outcome(s) of behaviour | Monitor and provide feedback on the outcome of performance of the behaviour. | 1 | 1 | 1 | 1 | 1 | 1 |
|  | *Social support* | | | | | | | |
| 3.1 | Social support (unspecified) | Advise on, arrange or provide social support (e.g. from friends, relatives, colleagues,’ buddies’ or staff) or non-contingent praise or reward for performance of the behaviour. It includes encouragement and counselling, but only when it is directed at the behaviour. |  |  |  |  | 1 |  |
| 3.2 | Social support (practical) | Advise on, arrange, or provide practical help (e.g. from friends, relatives, colleagues, ‘buddies’ or staff) for performance of the behaviour. |  |  |  |  |  |  |
| 3.3 | Social support (emotional) | Advise on, arrange, or provide emotional social support (e.g. from friends, relatives, colleagues, ‘buddies’ or staff) for performance of the behaviour. |  |  |  |  |  |  |
|  | *Shaping knowledge* | | | | | | | |
| 4.1 | Instruction on how to perform a behaviour | Advise or agree on how to perform the behaviour. | 1 |  |  | 1 |  |  |
| 4.2 | Information about antecedents | Provide information about antecedents that reliably predict performance of the behaviour. |  |  |  |  |  |  |
| 4.3 | Re-attribution | Elicit perceived causes of behaviour and suggest alternative explanations. |  |  |  |  |  |  |
| 4.4 | Behavioural experiments | Advise on how to identify and test hypotheses about the behaviour, its causes and consequences, by collecting and interpreting data. |  |  |  |  |  |  |
|  | *Natural consequences* | | | | | | | |
| 5.1 | Information about health consequences | Provide information (e.g. written, verbal, visual) about health consequences of performing the behaviour. | 1 | 1 |  |  | 1 | 1 |
| 5.2 | Salience of consequences | Use methods specifically designed to emphasise the consequences of performing the behaviour with the aim of making them more memorable (goes beyond informing about consequences). |  |  |  |  |  |  |
| 5.3 | Information about social and environmental consequences | Provide information (e.g. written, verbal, visual) about social and environmental consequences of performing the behaviour. |  |  |  |  |  |  |
| 5.4 | Monitoring of emotional consequences | Prompt assessment of feelings after attempts at performing the behaviour. |  |  |  |  |  | 1 |
| 5.5 | Anticipated regret | Induce or raise awareness of expectations of future regret about performance of the unwanted behaviour. |  |  |  |  |  |  |
| 5.6 | Information about emotional consequences | Provide information (e.g. written, verbal, visual) about emotional consequences of performing the behaviour. | 1 |  |  |  | 1 |  |
|  | *Comparison of behaviour* | | | | | | | |
| 6.1 | Demonstration of the behaviour | Provide an observable sample of the performance of the behaviour, directly in person or indirectly e.g. via film, pictures, for the person to aspire to or imitate (includes ‘Modelling’). | 1 |  |  | 1 |  |  |
| 6.2 | Social comparison | Draw attention to others’ performance to allow comparison with the person’s own performance. | 1 |  | 1 | 1 | 1 | 1 |
| 6.3 | Information about others’   approval | Provide information about what other people think about the behaviour. |  |  |  |  |  |  |
|  | *Associations* | | | | | | | |
| 7.1 | Prompts/cues | Introduce or define environmental or social stimulus with the purpose of prompting or cueing the behaviour. | 1 | 1 | 1 |  | 1 | 1 |
| 7.2 | Cue signalling reward | Identify an environmental stimulus that reliably predicts that reward will follow the behaviour. |  |  |  |  |  |  |
| 7.3 | Reduce prompts/cues | Withdraw gradually prompts to perform the behaviour. |  |  |  |  |  |  |
| 7.4 | Remove access to the reward | Advise or arrange for the person to be separated from situations in which unwanted behaviour can be rewarded in order to reduce the behaviour. |  |  |  |  |  |  |
| 7.5 | Remove aversive stimulus | Advise or arrange for the removal of an aversive stimulus to facilitate behaviour change. |  |  |  |  |  |  |
| 7.6 | Satiation | Advise or arrange repeated exposure to a stimulus that reduces or extinguishes a drive for the unwanted behaviour. |  |  |  |  |  |  |
| 7.7 | Exposure | Provide systematic confrontation with a feared stimulus to reduce the response to a later encounter. |  |  |  |  |  |  |
| 7.8 | Associative learning | Present a neutral stimulus jointly with a stimulus that already elicits the behaviour repeatedly until the neutral stimulus elicits that behaviour. |  |  |  |  |  |  |
|  | *Repetition and substitution* | | | | | | | |
| 8.1 | Behavioural practice/ rehearsal | Prompt practice or rehearsal of the performance of the behaviour one or more times in a context or at a time when the performance may not be necessary, in order to increase habit and skill. |  |  |  |  | 1 |  |
| 8.2 | Behaviour substitution | Prompt substitution of the unwanted behaviour with a wanted or neutral behaviour. |  |  |  |  |  |  |
| 8.3 | Habit formation | Prompt rehearsal and repetition of the behaviour in the same context repeatedly so that the context elicits the behaviour. |  |  |  |  | 1 |  |
| 8.4 | Habit reversal | Prompt rehearsal and repetition of an alternative behaviour to replace an unwanted habitual behaviour. |  |  |  |  |  |  |
| 8.5 | Overcorrection | Ask to repeat the wanted behaviour in an exaggerated way following an unwanted behaviour. |  |  |  |  |  |  |
| 8.6 | Generalisation of a target behaviour | Advise to perform the wanted behaviour, which is already performed in a particular situation, in another situation. |  |  |  |  |  |  |
| 8.7 | Graded tasks | Set easy-to-perform tasks, making them increasingly difficult, but achievable, until behaviour is performed. |  |  |  | 1 |  |  |
|  | *Comparison of outcomes* | | | | | | | |
| 9.1 | Credible source | Present verbal or visual communication from a credible source in favour of or against the behaviour. | 1 | 1 |  |  |  |  |
| 9.2 | Pros and cons | Advise the person to identify and compare reasons for wanting (pros) and not wanting to (cons) change the behaviour. |  |  |  |  |  |  |
| 9.3 | Comparative imagining of future outcomes | Prompt or advise the imagining and comparing of future outcomes of changed versus unchanged behaviour. |  |  |  |  |  |  |
|  | *Reward and threat* | | | | | | | |
| 10.1 | Material incentive (behaviour) | Inform that money, vouchers or other valued objects will be delivered if and only if there has been effort and/or progress in performing the behaviour. |  |  |  |  |  |  |
| 10.2 | Material reward (behaviour) | Arrange for the delivery of money, vouchers or other valued objects if and only if there has been effort and/or progress in performing the behaviour. |  |  |  |  |  |  |
| 10.3 | Non-specific reward | Arrange delivery of a reward if and only if there has been effort and/or progress in performing the behaviour. |  |  |  |  |  |  |
| 10.4 | Social reward | Arrange verbal or non-verbal reward if and only if there has been effort and/or progress in performing the behaviour. | 1 | 1 |  | 1 | 1 | 1 |
| 10.5 | Social incentive | Inform that a verbal or non-verbal reward will be delivered if and only if there has been effort and/or progress in performing the behaviour. | 1 |  |  | 1 | 1 |  |
| 10.6 | Non-specific incentive | Inform that a reward will be delivered if and only if there has been effort and/or progress in performing the behaviour. |  |  |  |  |  |  |
| 10.7 | Self-incentive | Plan to reward self in future if and only if there has been effort and/or progress in performing the behaviour. |  |  |  |  |  |  |
| 10.8 | Incentive (outcome) | Inform that a reward will be delivered if and only if there has been effort and/or progress in achieving the behavioural outcome. | 1 |  |  |  |  |  |
| 10.9 | Self-reward | Prompt self-praise or self-reward if and only if there has been effort and/or progress in performing the behaviour |  |  |  |  |  |  |
| 10.10 | Reward (outcome) | Arrange for the delivery of a reward if and only if there has been effort and/or progress in achieving the behavioural outcome. | 1 |  | 1 |  |  |  |
| 10.11 | Future punishment | Inform that future punishment or removal of reward will be a consequence of performance of an unwanted behaviour. |  |  |  |  |  |  |
|  | *Regulation* | | | | | | | |
| 11.1 | Pharmacological support | Provide, or encourage the use of or adherence to, drugs to facilitate behaviour change. |  |  |  |  |  |  |
| 11.2 | Reduce negative emotions | Advise on ways of reducing negative emotions to facilitate performance of the behaviour. |  |  |  |  |  |  |
| 11.3 | Conserving mental resources | Advise on ways of minimising demands on mental resources to facilitate behaviour change. |  |  |  |  |  |  |
| 11.4 | Paradoxical instructions | Advise to engage in some form of the unwanted behaviour with the aim of reducing motivation to engage in that behaviour. |  |  |  |  |  |  |
|  | *Antecedents* | | | | | | | |
| 12.1 | Restructuring the physical environment | Change, or advise to change the physical environment in order to facilitate performance of the wanted behaviour or create barriers to the unwanted behaviour. |  |  |  |  |  |  |
| 12.2 | Restructuring the social environment | Change, or advise to change the social environment in order to facilitate performance of the wanted behaviour or create barriers to the unwanted behaviour. |  |  |  |  |  |  |
| 12.3 | Avoidance/reducing exposure to cues for the behaviour | Advise on how to avoid exposure to specific social and contextual/physical cues for the behaviour, including changing daily or weekly routines. |  |  |  |  |  |  |
| 12.4 | Distraction | Advise or arrange to use an alternative focus for attention to avoid triggers for unwanted behaviour. |  |  |  |  |  |  |
| 12.5 | Adding objects to the environment | Add objects to the environment in order to facilitate performance of the behaviour. | 1 | 1 | 1 | 1 | 1 | 1 |
| 12.6 | Body changes | Alter body structure, functioning or support directly to facilitate behaviour change. | 1 | 1 | 1 | 1 | 1 | 1 |
|  | *Identity* | | | | | | | |
| 13.1 | Identification of self as role model | Inform that one's own behaviour may be an example to others. |  |  |  |  |  |  |
| 13.2 | Framing/reframing | Suggest the deliberate adoption of a perspective or new perspective on behaviour (e.g. its purpose) in order to change cognitions or emotions about performing the behaviour. |  |  |  |  |  |  |
| 13.3 | Incompatible beliefs | Draw attention to discrepancies between current or past behaviour and self-image, in order to create discomfort. |  |  |  |  |  |  |
| 13.4 | Valued self-identity | Advise the person to write or complete rating scales about a cherished value or personal strength as a means of affirming the person’s identity as part of a behaviour change strategy. |  |  |  |  |  |  |
| 13.5 | Identity associated with changed behaviour | Advise the person to construct a new self-identity as someone who ‘used to engage with the unwanted behaviour’. |  |  |  |  |  |  |
|  | *Scheduled consequences* | | | | | | | |
| 14.1 | Behaviour cost | Arrange for withdrawal of something valued if and only if an unwanted behaviour is performed. |  |  |  |  |  |  |
| 14.2 | Punishment | Arrange for aversive consequence contingent on the performance of the unwanted behaviour. |  |  |  |  |  |  |
| 14.3 | Remove reward | Arrange for discontinuation of contingent reward following performance of the unwanted behaviour. |  |  |  |  |  |  |
| 14.4 | Reward approximation | Arrange for reward following any approximation to the target behaviour, gradually rewarding only performance closer to the wanted behaviour. |  |  |  |  |  |  |
| 14.5 | Rewarding completion | Build up behaviour by arranging reward following final component of the behaviour; gradually add the components of the behaviour that occur earlier in the behavioural sequence. |  |  |  |  |  |  |
| 14.6 | Situation-specific reward | Arrange for reward following the behaviour in one situation but not in another. |  |  |  |  |  |  |
| 14.7 | Reward incompatible behaviour | Arrange reward for responding in a manner that is incompatible with a previous response to that situation. |  |  |  |  |  |  |
| 14.8 | Reward alternative behaviour | Arrange reward for performance of an alternative to the unwanted behaviour. |  |  |  |  |  |  |
| 14.9 | Reduce reward frequency | Arrange for rewards to be made contingent on increasing duration or frequency of the behaviour. |  |  |  |  |  |  |
| 14.10 | Remove punishment | Arrange for removal of an unpleasant consequence contingent on performance of the wanted behaviour. |  |  |  |  |  |  |
|  | *Self-belief* | | | | | | | |
| 15.1 | Verbal persuasion about capability | Tell the person that they can successfully perform the wanted behaviour, arguing against self-doubts and asserting that they can and will succeed. |  |  |  |  |  |  |
| 15.2 | Mental rehearsal of successful performance | Advise to practise imagining performing the behaviour successfully in relevant contexts. |  |  |  |  |  |  |
| 15.3 | Focus on past success | Advise to think about or list previous successes in performing the behaviour. |  |  |  |  |  |  |
| 15.4 | Self-talk | Prompt positive self-talk (aloud or silently) before and during the behaviour. |  |  |  |  |  |  |
|  | *Covert learning* | | | | | | | |
| 16.1 | Imaginary punishment | Advise to imagine performing the unwanted behaviour in a real-life situation followed by imagining an unpleasant consequence. |  |  |  |  |  |  |
| 16.2 | Imaginary reward | Advise to imagine performing the wanted behaviour in a real-life situation followed by imagining a pleasant consequence. |  |  |  |  |  |  |
| 16.3 | Vicarious consequences | Prompt observation of the consequences (including rewards and punishments) for others when they perform the behaviour. |  |  |  |  |  |  |
|  | *Total* |  | *20* | *11* | *11* | *13* | *19* | *12* |
